# A case of chronic elephantiasis

**DOI:** 10.11604/pamj.2024.47.74.41427

**Published:** 2024-02-20

**Authors:** Anjana Ledwani, Ashwin Karnan

**Affiliations:** 1 Department of Respiratory Medicine, Jawaharlal Nehru Medical College, Datta Meghe Institute of Higher Education and Research, Sawangi (Meghe), Wardha, Maharashtra, India

**Keywords:** Lymphatic filariasis, elephantiasis, lymphedema

## Image in medicine

A 72-year-old male presented with swelling over the left lower limb over the past 30 years, which has gradually increased in size in the past 5-6 years with the association of skin changes. The patient has been diagnosed with stage 4 lymphedema for 10 years and has been on a course of tablets of Diethylcarbamazine 100mg thrice daily for four weeks, which he repeats every six months. The patient now complains of increased pain and a burning sensation over the left foot, along with severe difficulty walking, for the past 15 days. Upon examination, the left leg is enlarged, non-pitting, and malodorous, and it has hard induration and hyperpigmented, cobblestone-like lesions that extend from the left foot to a few inches below the anterior superior iliac spine. The surrounding area was erythematous, warm to the touch, and tender. Multiple chronic, nonhealing ulcers with an erythematous base were seen on the posterior aspect of the ankle. The patient was febrile and the white blood cell count was raised (16,000/microliter). Lymphatic filariasis is a neglected tropical disease that is still rampant, affects 120 million people worldwide, and can lead to various complications in the chronic stage. The current patient presented to us in the advanced stage with chronic skin changes, non-healing ulcers, various papillomatous growths with secondary infection, and sepsis. Treatment modalities include chemotherapy with Diethylcarbamazine (DEC), control of superadded infection, wound care, and hygiene maintenance. Surgery can be done in extreme cases, which includes reconstructive surgery or surgical excision.

**Figure 1:**
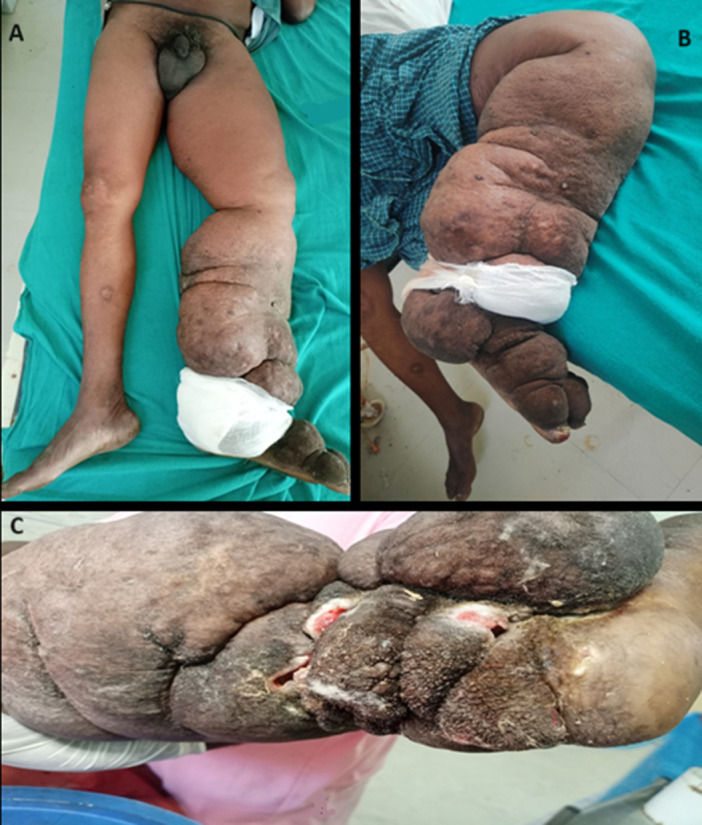
A) a comparison of both lower limbs showing lymphedema in the left lower limb along with scrotal edema while the right lower limb is normal; B) edematous left lower limb with hyperpigmentation and cobblestone-like lesions; C) the posterior aspect of the left ankle joint showing multiple non-healing ulcers with surrounding thickened and hyperpigmented skin

